# The Effect of Two Post-Space Preparation Techniques on the Seal of Resilon and Gutta-Percha Obturation Materials

**Published:** 2010-05-20

**Authors:** Kazem Ashofteh Yazdi, Hasan Razmi, Sholeh Ghabraei, Noushin Shokouhinejad, Marzieh Aligholi, Saeed Rahmani

**Affiliations:** 1. Department of Endodontics, School of Dentistry/Dental Research Center, Tehran University of Medical Sciences, Tehran, Iran.; 2. Department of Endodontics, School of Dentistry/Dental Research Center, Tehran University of Medical Sciences, and Member of Iranian Center for Endodontic Research, Tehran, Iran.; 3. Department of Microbiology, School of Medicine, Tehran University of Medical Sciences, Tehran, Iran.; 4. Department of Restorative Dentistry, Dental School, Kerman University of Medical Sciences, Kerman, Iran.

**Keywords:** Gutta-Percha, Leakage, Post Space, Resilon, Solvents

## Abstract

**INTRODUCTION:**

The aim of this study was to compare the effect of post space preparation with Gates Glidden drills or hand files on the sealing ability of gutta-percha or Resilon obturation materials.

**MATERIALS AND METHODS:**

One-hundred and four single-rooted human teeth were instrumented and divided into four experimental groups (n=21 each) and two groups of positive and negative control (n=10). Forty-two roots of experimental groups were obturated with gutta-percha and Dorifill sealer; and 42 roots with Resilon/Epiphany self-etch using lateral compaction technique. Each gutta-percha or Resilon group was divided into two subgroups (n=21) and post-space prepared with either Gates Glidden drills or hand files and chloroform. After post space preparation, 5 mm of apical gutta-percha or Resilon was left intact. The whole system was sterilized with gamma-rays. Saliva leakage was tested using a split-chamber model. Specimens were monitored every 24 hours for 30 days. The data were analyzed using log-rank test and Cox regression analysis.

**RESULTS:**

There were no significant differences between the sealing ability of gutta-percha and Resilon. Furthermore, no significant difference was found between two different methods of post space preparation (P>0.05).

**CONCLUSION:**

Under the limitations of this ex vivo study, there were no significant differences between the sealing ability of Resilon and gutta-percha after post space preparation using Gates Glidden drills or hand files with the aid of chloroform.

## INTRODUCTION

Post space preparation techniques are important for several reasons. One concern is the maintenance of the obturation seal which averts microbial invasion, and therefore prevents the possibility of endodontic failure [1].

Resilon is a bonded root canal obturation system and in theory it should provide a superior seal compared to gutta-percha after post space preparation. According to Shipper et al. [2], bacterial microleakage of Resilon is significantly less than that of gutta-percha when used as the root canal filling material. However, other studies did not find significant difference between the sealing ability of Resilon and gutta-percha [3][4][5].

There are various methods for preparing post-space such as mechanical (bur/drills), physical (heated instruments) or chemical (solvents) techniques [6][7]. During preparation the remaining filling material might be dislocated, which can create a pathway for re-infection of the root canal system [1]. Therefore, selecting an appropriate root canal filling material and procedure for post space preparation is an essential factor that can affect the treatment outcome. It is arguable whether post space preparation with solvents such as chloroform interfere with the apical seal of a root canal, though solvents have been shown to have unpredictable penetration [8], other studies reported no significant difference among the post space preparation techniques (heated plugger, LA Axxess burs, and hand files with the aid of chloroform) in terms of bacterial leakage from remaining gutta-percha/AH Plus in the apical area [1].

Bodrumlu et al. showed significant difference in microleakage between gutta-percha/AH-Plus and Resilon/Epiphany-filled groups after immediate mechanical post space preparation [9]. Similarly, Muñoz et al. studied the penetration of Enterococcus (E) faecalis and showed no significant difference between the seal of gutta-percha and Resilon after immediate post space preparation [10].

The aim of the present study was to compare the sealing ability of gutta-percha/Dorifill sealer and Resilon/Epiphany SE obturation materials against penetration of human saliva when the canals had post spaces preparations with either (i) Gates Glidden drills or (ii) hand files + chloroform.

## MATERIALS AND METHODS

One hundred and four single-rooted human teeth with the absence of caries or fractures were selected. The teeth were disinfected with NaOCl and then decoronated to obtain root length of 15 mm. The canals were prepared with K-files using passive step back technique to the apical size #30. The root canals were irrigated after each file with 3 mL of NaOCl 5.25%. Finally, root canals were irrigated with 3 mL of EDTA 17% for 1 minute, to remove smear layer. Then the specimens were divided into four experimental groups (n=21) and two groups of negative and positive controls (n=10). Forty-two teeth were obturated with gutta-percha (Gapadent Co., Ltd., Korea) and Dorifill sealer (Dorident Co., Austria), and 42 teeth with Resilon/Epiphany self-etch (Pentron Clinical Technologies, LLC. Wallingford, CT) using the lateral compaction technique. After radiographic confirmation of the obturation quality, the coronal part of each root was sealed with a temporary filling material and teeth were placed in an incubator under 37˚C and 100% humidity for one week. The root canals in positive control group were filled with a single cone of gutta-percha (#30) without sealer, and in negative control group with gutta-percha/Dorifill sealer in the same manner as the experimental groups.

Experimental groups were divided into 2 subgroups (n=21) as follows:

Group 1: the samples were obturated with gutta-percha/Dorifill and post spaces were prepared with Gates Glidden drills (#2, 3, and 4) using step-back technique.

Group 2: the samples were obturated with gutta-percha/Dorifill, and post space preparation was performed with the aid of chloroform and hand K-files.

Group 3: the samples were obturated with Resilon/Epiphany SE and post spaces were prepared with Gates Glidden drills.

Group 4: the samples were obturated with Resilon/Epiphany SE and post space preparations were performed with chloroform and hand K-files.

Groups 1 and 3 post space preparation was prepared with Gates Glidden drills no #2, 3, and 4; 5 mm of apical gutta-percha or Resilon was left intact. For post space preparation with the aid of solvent, 2 mm of Resilon or gutta-percha was removed with a hot carrier in order to prepare a small pit for applying the solvent. Then, 2-3 drops of chloroform were dispensed from an insulin syringe in to each canal. Then post space was prepared with stainless steel K-files (#15 to #60). A total of 0.3 mL chloroform was used in each tooth. Finally, radiographic images were obtained from the specimens to confirm complete removal of filling material from coronal segment of canals. All procedures were performed by a single operator.

All the teeth in experimental and positive control groups received two layers of nail varnish (Arcancil, Paris, France) except for the root’ apical 3 mm. Negative control specimens were coated completely with two layers of nail varnish.

A split-chamber model was used for bacterial leakage evaluation. The taper end of the 2-mL plastic Eppendorf tube was cut and each root was placed into the tube. The root was positioned inside the tube so that its apical end was removed from the cut-end section of the Eppendorf tube. The junction between the plastic tube and the root was sealed with sticky wax. The apparatus (teeth and plastic tubes) were sterilized by exposure to 40 k-Gray Gamma irradiation. The specimens were incubated at 37˚C for 3 days to confirm sterility of the system. Then the Eppendorf tube containing the samples was placed in a glass tube with thioglycolate broth, so that at least 2 mm of the root apex was immersed in the broth. The junction between the Eppendorf and the glass tube was sealed tightly with sticky wax. For confirming the sterility of system, samples were kept for 3 days under 37˚C in an incubator. If turbidity was observed in the thioglycolate broth, the sample was sterilized again. Subsequently, the upper chamber of the split-model was filled with human saliva. The saliva was changed every 3 days. The samples were incubated at 37˚C and evaluated daily for the existence of turbidity in the thioglycolate broth in the lower chamber of the system. To confirm identical bacterial contamination in both the upper and lower chambers, cultures from the lower chamber were streaked onto blood agar culture plates and incubated under aerobic and anaerobic conditions at 37˚C.

Survival analysis and log-rank testing compared the survival curve patterns of the experiment. Kaplan-Meier survival curves were constructed based on the leakage of specimens over time. Cox regression analysis was used in testing the interaction between root canal filling materials and post space preparation methods. The significance level was set at P=0.05.

## RESULTS

All samples in the positive control group showed broth turbidity within 3 days of the incubation. The negative controls prevented leakage for the entire 30 day experimental period.

**Figure 1:**
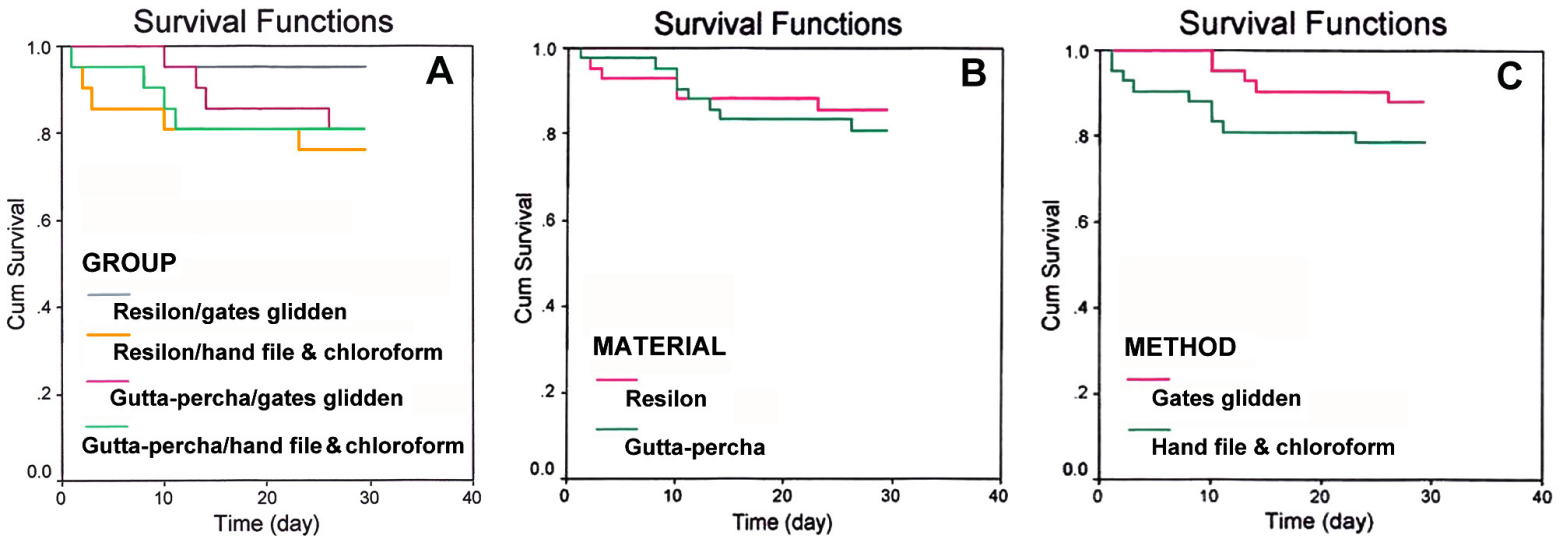
Kaplan-Meier survival curve considering A) the experimental groups, B) root canal filling materials, and C) post space preparation methods

The canal obturation material, post space preparation methods and the time of leakage were all statistically compared. The Kaplan-Meier curves are shown in Figure 1A.

***Comparison of microleakage of the different obturation materials without considering post space preparation methods:*** 8 samples (19%) in gutta-percha groups (n=42) and 6 samples (14.3%) in Resilon groups (n=42) leaked after 30 days (Figure 1B). The difference was not statistically significant (P=0.4).

***Comparison of microleakage between the two post space preparation technique without considering obturation materials:*** The hand file + chloroform groups (n=42), 9 samples (21.4%), and the Gates Glidden groups (n=42) had 5 samples (11.9%) leaked after 30 days (Figure 1C). The difference was not statistically significant. (P=0.25).

Numbers of leaked samples at the end of a 30- day period are shown in Table 1.

**Table 1:** Number (percentage) of positive leakage samples at the end of a 30-day period

** Group **	** Number (%) of positive leakage samples **
Group 1 (n=21)	4 (19%)
Group 2 (n=21)	4 (19%)
Group 3 (n=21)	1 (4.8%)
Group 4 (n=21)	5 (23.8%)

## DISCUSSION

This study compared the polymicrobial microleakage of Resilon and gutta-percha using human saliva. The sterilization of samples was performed with gamma-rays as there are reports that gamma irradiation is one of the best forms of sterilization [11][12].

In the present study, there were no significant differences between remaining Resilon and gutta-percha obturation sealing ability after post space preparation. We used human saliva to stimulate the in vivo environment. Our results are in agreement with De-Deus et al. who used human saliva and did not find significant difference between gutta-percha and Resilon [4]. Shipper et al. [2] reported that microleakage of Streptococcus mutans and E. faecalis through Resilon was significantly lower compared to gutta-percha when used as the root filling materials. However, Baumgartner et al. [13] showed that there was no significant difference between the sealing ability of Resilon and gutta-percha against E. faecalis. The different methodologies for evaluating sealing ability may have resulted in the different findings.

In this study, the microleakage of remaining gutta-percha was not different after post space preparation with rotary instruments (Gates Glidden) or hand files + chloroform. Similarly, Grecca et al. [1] found that there was no difference among the post space preparation techniques (heated plugger, LA Axxess burs, and hand files + chloroform) in terms of bacterial leakage through the gutta-percha/AH Plus root filling material.

Lyons et al. [14] also showed that the sealing ability of Resilon and gutta-percha was not significantly different after immediate or delayed post space preparation. Similarly, Muñoz et al. [10] found that the microleakage of E. faecalis was not different among remaining Resilon and gutta-percha after immediate post space preparation. However, these two studies [10][14] focused on the time of post space preparation rather than the methods for preparing the dowel space. Currently, there are no published articles that compare techniques for post space preparation in teeth obturated with gutta-percha or Resilon/Epiphany SE.

Moreover, this was the first study that used the new generation of Epiphany, which is self-etching (SE). The self-etch (self adhesive) types of methacrylate resin-based sealers have eliminated the use of separate self-etching primers by incorporating acidic resin monomers in the sealers [15].

## CONCLUSION

Under the conditions of this ex vivo study, there were no significant difference between the sealing ability of Resilon and gutta-percha after post space preparation with or without the aid of chloroform. However, further clinical studies should be designed to confirm these results.
